# Design and Evaluation of a Polypeptide That Mimics the Integrin Binding Site for EDA Fibronectin to Block Profibrotic Cell Activity

**DOI:** 10.3390/ijms22041575

**Published:** 2021-02-04

**Authors:** Lin Zhang, Hongyu Yan, Yifan Tai, Yueming Xue, Yongzhen Wei, Kai Wang, Qiang Zhao, Shufang Wang, Deling Kong, Adam C. Midgley

**Affiliations:** 1 Key Laboratory of Bioactive Materials, Ministry of Education, College of Life Sciences, Nankai University, Tianjin 300071, China; 1120180455@nankai.edu.cn (L.Z.); 1120190538@nankai.edu.cn (H.Y.); 1120190539@nankai.edu.cn (Y.T.); 2120181163@nankai.edu.cn (Y.X.); 1120170431@nankai.edu.cn (Y.W.); 013053@nankai.edu.cn (K.W.); qiangzhao@nankai.edu.cn (Q.Z.); kongdeling@nankai.edu.cn (D.K.); 2 Rongxiang Xu Center for Regenerative Life Science, State Key Laboratory of Medicinal Chemical Biology, College of Life Sciences, Nankai University, Tianjin 300071, China

**Keywords:** blocking peptide, molecular docking, fibrosis, antifibrotic, EDA fibronectin, integrin α4β1, fibroblast, myofibroblast

## Abstract

Fibrosis is characterized by excessive production of disorganized collagen- and fibronectin-rich extracellular matrices (ECMs) and is driven by the persistence of myofibroblasts within tissues. A key protein contributing to myofibroblast differentiation is extra domain A fibronectin (EDA-FN). We sought to target and interfere with interactions between EDA-FN and its integrin receptors to effectively inhibit profibrotic activity and myofibroblast formation. Molecular docking was used to assist in the design of a blocking polypeptide (antifibrotic 38-amino-acid polypeptide, AF38Pep) for specific inhibition of EDA-FN associations with the fibroblast-expressed integrins α_4_β_1_ and α_4_β_7_. Blocking peptides were designed and evaluated in silico before synthesis, confirmation of binding specificity, and evaluation in vitro. We identified the high-affinity EDA-FN C-C′ loop binding cleft within integrins α_4_β_1_ and α_4_β_7_. The polypeptide with the highest predicted binding affinity, AF38Pep, was synthesized and could achieve specific binding to myofibroblast fibronectin-rich ECM and EDA-FN C-C′ loop peptides. AF38Pep demonstrated potent myofibroblast inhibitory activity at 10 µg/mL and was not cytotoxic. Treatment with AF38Pep prevented integrin α_4_β_1_-mediated focal adhesion kinase (FAK) activation and early signaling through extracellular-signal-regulated kinases 1 and 2 (ERK1/2), attenuated the expression of pro-matrix metalloproteinase 9 (MMP9) and pro-MMP2, and inhibited collagen synthesis and deposition. Immunocytochemistry staining revealed an inhibition of α-smooth muscle actin (α-SMA) incorporation into actin stress fibers and attenuated cell contraction. Increases in the expression of mRNA associated with fibrosis and downstream from integrin signaling were inhibited by treatment with AF38Pep. Our study suggested that AF38Pep could successfully interfere with EDA-FN C-C′ loop-specific integrin interactions and could act as an effective inhibitor of fibroblast of myofibroblast differentiation.

## 1. Introduction

Fibrosis is a disease process characterized by excessive and disorganized extracellular matrix (ECM) deposition that leads to organ dysfunction and, in some cases, organ failure and death [1,2]. The principal cell types that contribute to fibrogenesis and the onset of fibrosis are α-smooth muscle actin (α-SMA) stress fiber-positive and contractile myofibroblasts [3,4]. Myofibroblast-mediated progressive fibrosis can befall most organs and tissues, giving rise to multiple end-stage organ diseases, including scleroderma [5], myocardial infarction [6], liver cirrhosis [7], pulmonary disease [8], and chronic kidney disease [9], among others. The fibrotic ECM is comprised of large amounts of activated fibroblast- and myofibroblast-synthesized collagen I; fibronectin (FN); the alternatively spliced variant of FN, extra domain A containing fibronectin (EDA-FN), and the glycosaminoglycan hyaluronan [10]. The classical view is that for activated fibroblasts to differentiate into myofibroblasts, transforming growth factor-β1 (TGF-β1) and a mechanically stiff ECM containing EDA-FN are necessary precursors and requirements [2,4,11,12].

The protein structure of fibronectin consists of twelve type I domains, two type II domains, and fifteen type III domains containing the two alternatively spliced extra domain A (EDA) and extra domain B (EDB) domains, and a connecting segment (IIICS) that has a variable expression [13,14]. Cellular fibronectin that undergoes alternative splicing to include EDA is synthesized by fibroblasts, endothelial cells, myoblasts [15], and osteoblasts [16]; yet EDA-FN splice variant only appears to be expressed with implications in wound repair [17,18], pathological fibrosis [19], and tumor development [20,21,22]. EDA-FN is difficult to detect in normal adult tissues and is only transiently expressed during embryogenesis [23]. Although uninjured adult EDA-FN-null mice are indistinguishable from wild-type mice, their injured counterparts demonstrated abnormal skin wound healing, ulcerations, and elevated inflammation at sites of injury [18]. Persistent accumulation of EDA-FN isoforms is observed in a range of human fibrotic disorders such as pulmonary fibrosis [19], liver fibrosis [22], keloid scars [24], and scleroderma [25]. Mouse genetic knockout EDA-FN-null models do not develop pulmonary fibrosis after bleomycin insult and showed reduced numbers of activated fibroblast to myofibroblast differentiation [26]. These findings demonstrated the indispensable roles that EDA-FN plays in the pathogenesis of fibrosis, and that its expression advances activated fibroblast to myofibroblast differentiation.

The detailed cellular mechanisms by which EDA-FN facilitates fibrotic responses have not yet been fully elucidated, but evidence suggests that interactions between latent TGF-β1 activation [27], EDA-FN-dependent increases in matrix stress–strain properties [28,29], and the activation of certain mechanotransducer integrin receptors to trigger downstream signaling and transcriptional activity [30,31,32] cumulate and drive the myofibroblast differentiation process. It is without doubt that fibronectin association with cells is complex, binding a string of cell surface receptors via a multitude of binding domains. The integrin family are transmembrane dimeric receptors that transmit mechano-transduction signals from the extracellular matrix (ECM) to the intracellular cytoskeleton and vice versa, to drive both cellular and ECM remodeling responses. The type III domains of FN contain RGD and PHSRN amino acid motifs that can bind FN-recognition sites within integrins α_5_β_1_, α_IIb_β_3_, α_v_β_3_ [33] and α_3_β_1_ [34], to promote cell adhesion and activate integrin signaling that drives FN filament assembly [35,36,37]. In the context of fibrogenesis, research by Shinde et al. [38] and Kohan et al. [32] on EDA-FN/integrin interactions demonstrated that the fibroblast-expressed integrins α_4_β_1_ (also known as VLA-4) and α_4_β_7_ (also known as LPAM-1) played different but related roles in mediating fibroblast to myofibroblast formation, respectively. Integrin α_4_β_7_-mediated EDA-FN binding induced myofibroblast-associated stress fiber formation and thus, contractility in murine lung fibroblasts [32], whereas integrin α_4_β_1_ activation by EDA-FN promoted profibrotic ECM synthesis and subsequent matrix stiffening in human dermal fibroblasts [38]. Therefore, signaling transduction through both α_4_β_7_ and α_4_β_1_ are key events that ultimately lead to mature myofibroblast transdifferentiation. The research identified that the integrin interactions with the EDGIHEL motif, which is exclusively located within a polypeptide exposed loop at the EDA C′-C region, was causative of downstream profibrotic responses. In addition, it was suggested that recombinant fragments of EDA could be used to interfere with and block EDA-FN induction of myofibroblast activation [28]. However, it was later shown that binding of recombinant EDA-FN C-C′ loop peptides to integrin α_4_β_1_ was sufficient to reproduce the fibrotic response [38] and may indicate that peptides derived from EDA provoke myofibroblast differentiation responses under pathological conditions.

The antibodies IST-9 [28,29] and F8 [39,40] can target EDA-FN and mask a large portion of protein from its interaction sites, essentially resulting in off-target blockade of functionality. Therapeutic vaccination against EDA-FN produced by stromal myofibroblasts was shown to attenuate the progression of metastatic breast cancer [41]. Whilst antibodies have proven effective in preventing fibroblast to myofibroblast differentiation [28,29] and in the antagonistic blockade of integrin α_4_ subunit-mediated activities [42], their use as a therapeutic option is limited given the cost and methodology of their production, risk of immune activation, and risk of off-target effects attributed to the large quaternary structure and size (~150 kDa) of antibodies interfering with critical protein–protein interactions. Small blocking polypeptides offer an attractive alternative and can be designed to bind and block regions of proteins with high specificity. Small peptides can also be synthesized at a decreased cost, have long-term stability, and can increase efficacy of a variety of treatment options. For example, peptides can be easily incorporated into biomaterials, or conjugated to bioimaging probes for more complex therapeutic usage. Therefore, this investigation aimed to use in silico molecular docking analysis to identify the EDA-FN C-C′ loop’s binding regions on a newly generated integrin α_4_β_1_ receptor model and on the resolved α_4_β_7_ receptor model. The new affinity and contact data were then used to design polypeptides that mimic the receptor binding site and block any EDA-FN C-C′ loop interactions with its cellular receptors. The resultant blocking polypeptide with the highest binding affinity (named AF38Pep) was assessed for potential to attenuate TGF-β1 stimulated myofibroblast formation, block integrin α_4_β_1_ signaling, and prevent the subsequent induction of profibrotic genes. The advantage of using blocking peptides in this way is to allow for the interference of ligand site-specific interactions with receptors, without disrupting the numerous other conveyed functions of EDA-FN and FN. Overall, we showed that our EDA-FN blocking peptide attenuated EDA-FN/integrin α_4_β_1_-mediated myofibroblast differentiation and profibrotic ECM generation, revealing an accessible candidate for effective antifibrotic therapies.

## 2. Results

### 2.1. Molecular Docking to Predict the Integrin α_4_β_1_/β_7_ Receptor Biding Site for EDA-FN

**Figure 1:**
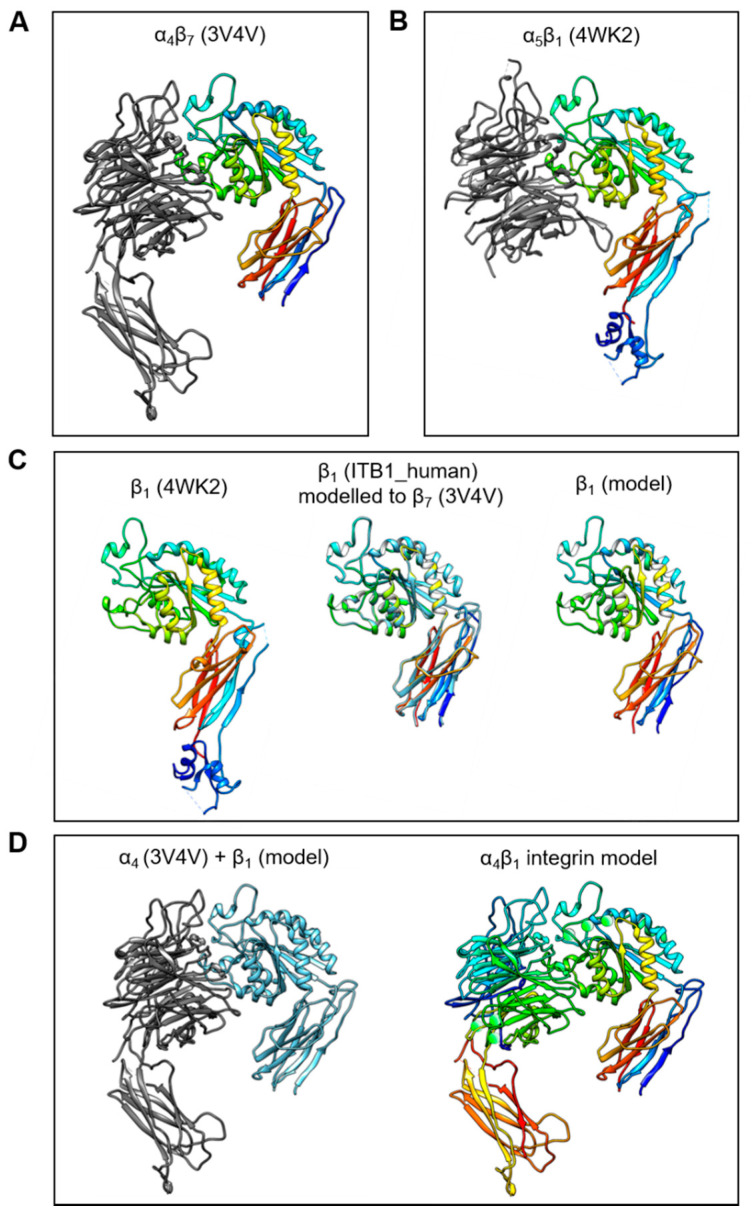
Generation of the integrin α_4_β_1_ protein model. The process of generating a working integrin α_4_β_1_ receptor protein model was as follows: (**A**) The α_4_β_7_ resolved head domain model (PDB code 3V4V) and (**B**) the α_5_β_1_ resolved head domain model (PDB code 4WK2) were utilized as templates for α_4_ and β_1_ subunits, respectively. (**C**) The β_1_ subunit (4WK2) was mapped using the ITB1_human peptide sequence and the β_7_ subunit (3V4V) as references, to generate a β_1_ subunit model in active conformation. (**D**) The α_4_ (3V4V) and newly generated β_1_ model subunits (left image) were compiled into a single α_4_β_1_ integrin model (right image) with H-bonds and metal ions (green spheres).

The C-C′ loop of the EDA-FN protein has been shown to interact with integrin α_4_β_1_ and integrin α_4_β_7_ [32,38]. Integrin α_4_β_7_ has been resolved as a 3D crystal structure; however, α_4_β_1_ has not yet been resolved. Therefore, in order to predict and compare receptor binding sites, an integrin α_4_β_1_ model was required. To generate a working integrin α_4_β_1_ model, the resolved structures for α_4_β_7_ (3V4V; Figure 1A) and α_5_β_1_ (4WK2; Figure 1B) were obtained from the PDB online repository. The integrin β_1_ (ITB1_human) subunit shares 42.112% amino acid sequence homology with the integrin β_7_ (ITΒ7_human) subunit. Thus, the ITB7_human amino acid sequence was used as a reference sequence and the 3D structure of the integrin β_7_ subunit from the α_4_β_7_ model was used as a template upon which the integrin β_1_ subunit was mapped (Figure 1C). A structural comparison with the β_1_ subunit conformation and orientation taken from the α_5_β_1_ model showed that the new β_1_ subunit model had protein structural domains within similar, if not identical, regions of the subunit. Next, the α_4_ subunit from the α_4_β_7_ model, and the new β_1_ subunit model, were compiled into a single receptor model (Figure 1D). H-bonds and metal ions were reassigned before validation using PROCHECK and Verify3D protein model analysis software.

**Figure 2:**
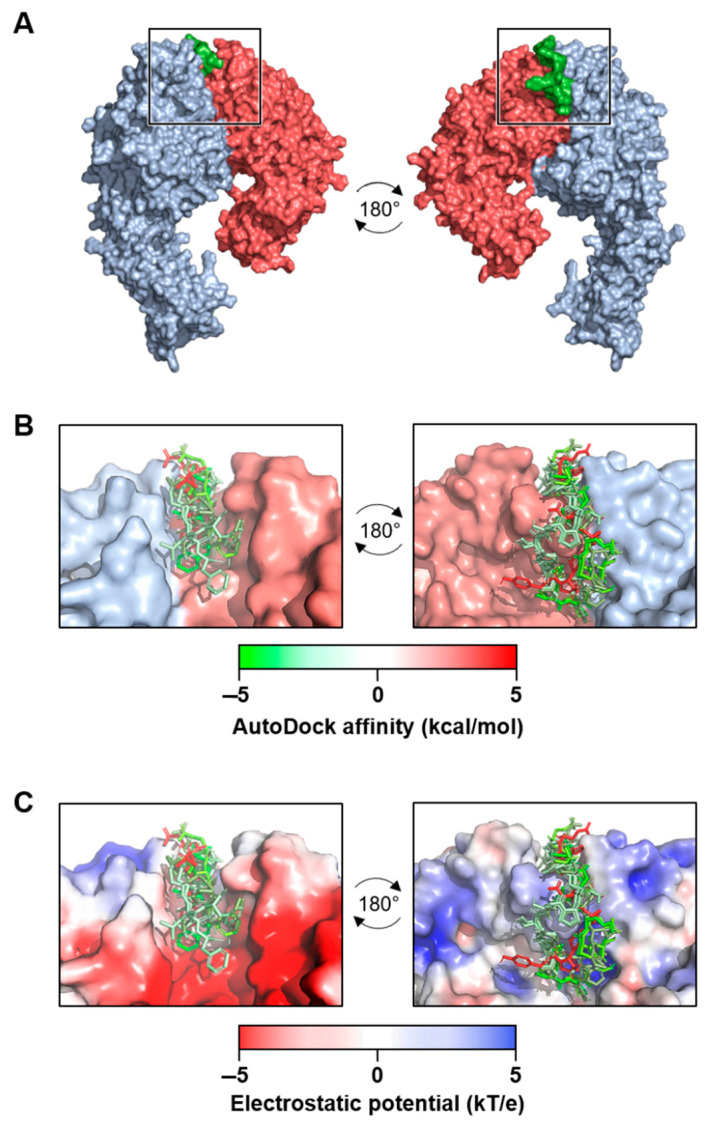
Molecular docking analysis of extra domain A fibronectin (EDA-FN) peptide domain binding to the α_4_β_1_ receptor model. (**A**) The –TYSSPEDGHIEL-- peptide domain of the EDA-FN protein (green) was utilized as a ligand for molecular docking analysis to the integrin α_4_ (blue) β_1_ (red) receptor model. From the areas of the receptor analyzed, only the receptor binding cleft (boxed region) returned valid affinity score values. A 180° rotation is shown in order to visualize the peptide within the receptor binding cleft. (**B**) The top 7 binding modes of the EDA-FN peptide within the integrin receptor binding cleft. Green (–5) to red (+5) gradient colorization relates to the AutoDock affinity (kcal/mol) scores. (**C**) The electrostatic potential (kT/e) of the receptor binding cleft was predicted and is shown as a red (–5) to blue (+5) gradient. Note that the peptide had an overall stronger binding mode in the positive region of the binding cleft.

**Figure 3:**
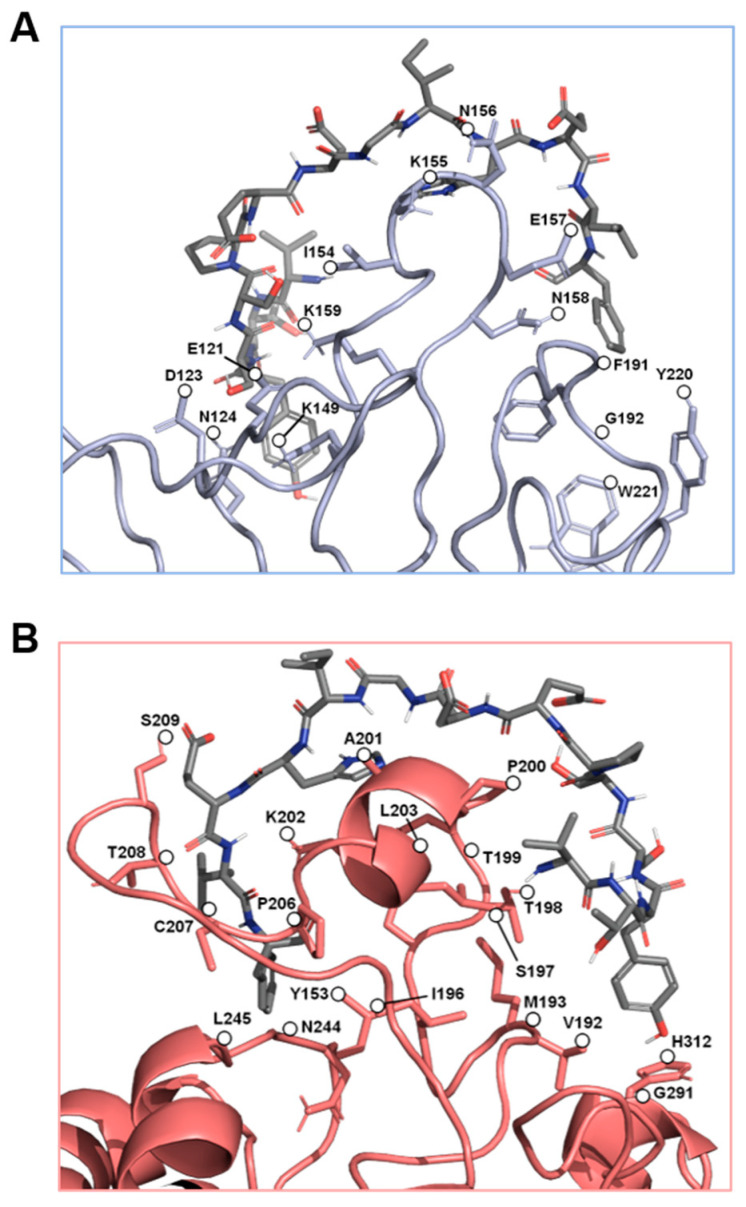
The predicted key binding amino acids of the triple loop structures from the α_4_ and β_1_ subunits. The amino acids with the highest predicted binding affinities to the EDA-FN peptide (grey) are highlighted within (**A**) the α_4_ subunit triple loop finger-like domains of the integrin receptor cleft (blue), and (**B**) the β_1_ subunit triple loop finger-like domains of the integrin receptor cleft (red). The β_1_ subunit was used for visualization.

The α_4_β_7_ model and the new α_4_β_1_ receptor model were examined for potential binding of the C-C′ loop of EDA-FN. Across five separate grid analyses, which altogether covered the entirety of the receptor models’ surfaces, only the grid that contained the head of the receptor binding cleft returned viable binding affinity data for both integrin receptors (Figure 2A). The top seven binding modes all had similar binding orientations and were aligned in the same directionality (Figure 2B). Binding mode AutoDock affinity scores ranged from between −5 to 5 kcal/mol, with 6 of the 7 modes falling within the negative range, suggesting stronger indications for ligand binding at this receptor site. An assessment of the receptors’ electrostatic potentials (Figure 2C) indicated that the majority of the ligand chain tended towards the positively charged regions (0–5 kT/e) of the receptor binding cleft, which suggests stronger electrostatic associations between the amino acids of the ligand and the amino acids of the receptor binding cleft. The amino acids within 3 Å of contact distance were calculated and potential hydrogen bond donors were noted (Table 1). The data showed the amino acids with the strongest predicted binding potentials and suggested that shared EDA-FN binding motifs were present within both integrin β_1_ and β_7_ subunits. Figure 3 shows the EDA-FN peptide within 3 Å contact distance with the predicted binding amino acids of integrin α_4_ (Figure 3A) and integrin β_1_ (Figure 3B). These results provided sufficient evidence and information from which to base the design of peptides that mimic the integrin binding site for the C-C′ loop of EDA-FN.

### 2.2. Modeling and Synthesis of EDA-FN Blocking Polypeptide

**Figure 4:**
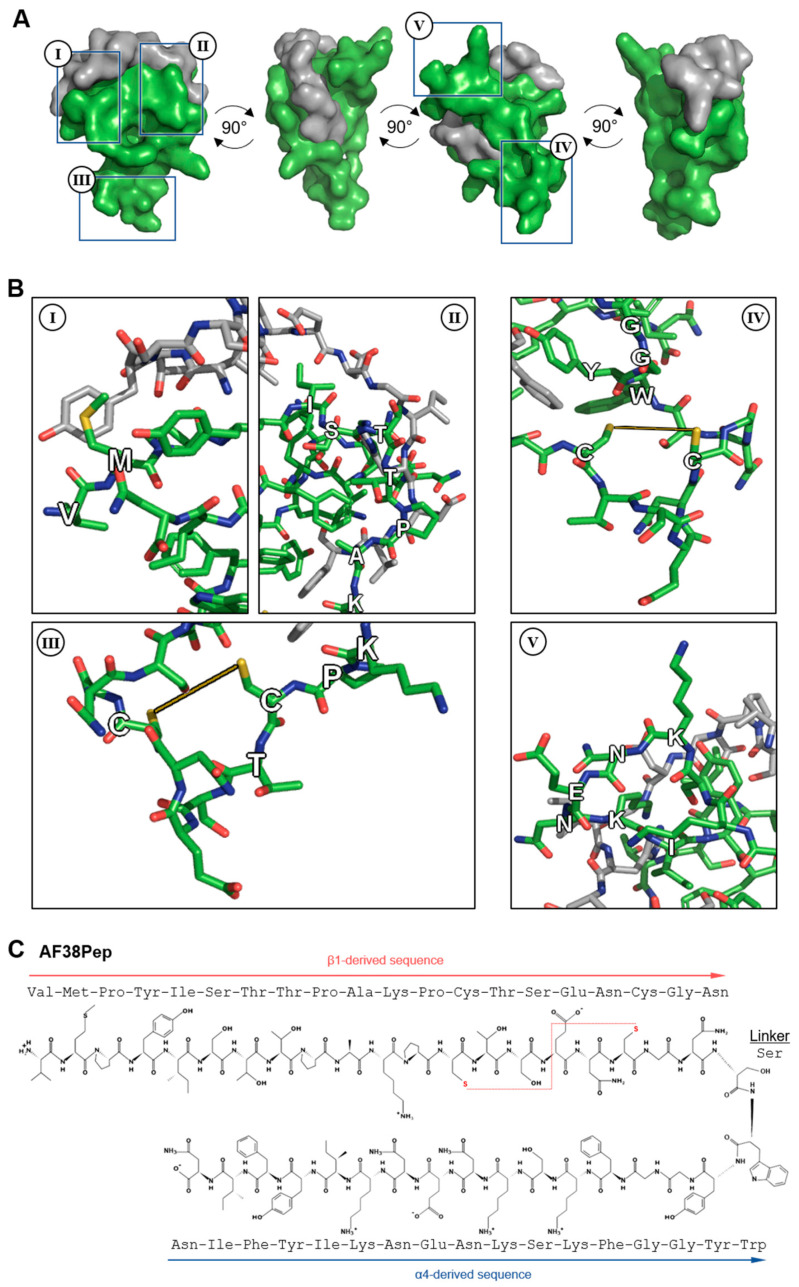
Molecular docking analysis of the designed blocking polypeptide and key features. (**A**) The EDA-FN peptide (grey) was docked to the blocking polypeptide (green) derived from the polypeptide with the overall highest scores across all refinement analyses. A 360° rotation is shown to visualize the wrapping and binding of the EDA-FN peptide to the blocking polypeptide, which mimics the integrin α_4_β_1_/β_7_ receptor binding site. Boxed regions I–IV are shown in (**B**) to highlight the key features of the designed blocking polypeptide. I, the VM cap; II, the ISTTPAK motif of the β_1_/β_7_ subunit; III and IV, the C=C disulfide bridge to form the receptor and cage-like conformation; V, the KNENKI motif of the α4 subunit. (**C**) The antifibrotic 38-amino-acid polypeptide (AF38Pep) polypeptide sequence structure, showing two peptide chains derived from integrins β_1_ and α_4_, linked by a serine. Disulfide bridge is shown by a red dashed line, bridging the two cysteines.

Based on the results from Table 1 and Figure 3, a series of candidate blocking polypeptides were designed and evaluated through stringent assessment by peptide folding and molecular docking. The best performing polypeptide (Figure 4A) was docked with the C-C′ loop EDA-FN peptide and demonstrated a binding score of ≤−5 kcal/mol, suggesting strong binding potential. The designed peptide candidate had a cage-like conformation to mimic the integrin α_4_β_1_ receptor binding cleft and the 3 finger-like protrusions from each subunit that had the highest interactivity with the EDA-FN peptide. Key features of the designed polypeptide are shown in Figure 4B(I–V). The valine-methionine cap (Figure 4B(I)) had the highest binding affinity to the 5′ end of the C-C′ loop of EDA-FN and the upstream amino acids. The ISTTPAK motif from the β_1_/β_7_ subunit (Figure 4B(II)) showed the most consistent representation across all predicted binding modes. A cysteine disulfide bridge was incorporated (Figure 4B(III,IV)) to provide a folded apex, which allowed for the α_4_- and β_1_-like cages to flank either side of the EDA-FN peptide. The KNENKI motif from the α_4_ subunit (Figure 4B(V)) was included to mimic the most reoccurring binding motif across predicted binding modes. Thus, this candidate antifibrotic 38-amino-acid polypeptide (AF38Pep) design (Figure 4C; sequence, VMPYISTTPAKPCTSENCGNSWYGGFKSKNENKIYFIN) was chosen for synthesis and subsequent testing using in vitro assays.

### 2.3. Evaluation of Polypeptide Cytocompatibility and Antifibrotic Function

**Figure 5:**
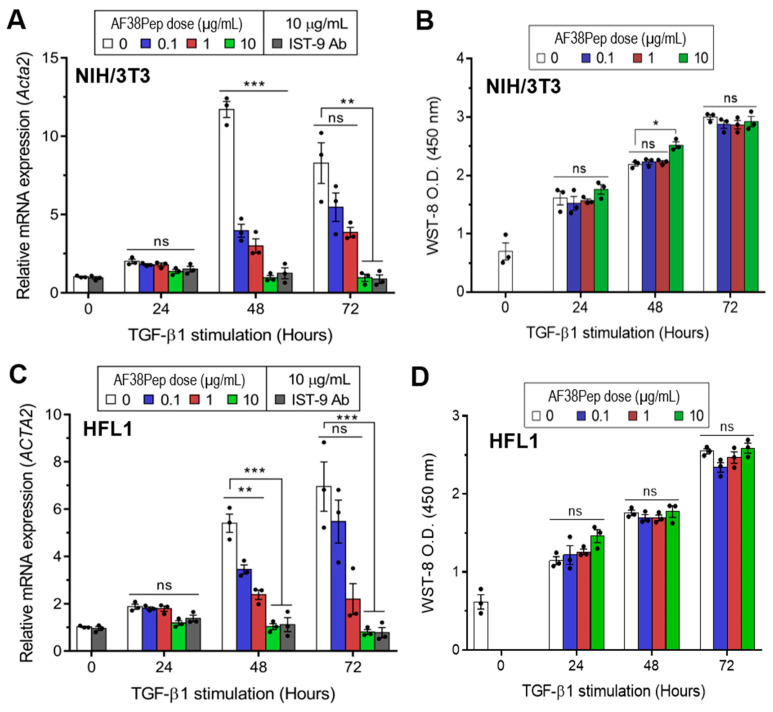
Cytocompatibility characteristics of the synthesized blocking polypeptide. (**A**) The ability for AF38Pep to attenuate transforming growth factor-β1 (TGF-β1) induced upregulation in expression of the myofibroblast marker, α-smooth muscle actin (α-SMA), in mouse NIH/3T3 dermal fibroblast cells over 72 h. (**B**) Cytotoxicity/proliferation assays in TGF-β1 stimulated mouse NIH/3T3 dermal fibroblast cells over 72 h. (**C**) The ability for AF38Pep to attenuate TGF-β1 induced upregulation in expression of α-SMA in human HFL1 lung fibroblast cells over 72 h. (**D**) Cytotoxicity/proliferation assays in TGF-β1 stimulated human HFL1 lung fibroblast cells over 72 h. IST-9 antibody was used as a positive control for EDA-FN blockade. Data are representative of three independent experiments and displayed as the mean ± S.E. Statistical analysis is shown as *** *p* ≤ 0.001, ** *p* ≤ 0.01, * *p* ≤ 0.05, and ns = no statistical significance (*p* > 0.05).

To determine whether the blocking polypeptide had antifibrotic functionality or exhibited cytotoxicity, we incubated TGF-β1-treated mouse dermal fibroblasts (NIH/3T3; Figure 5A,B) or human lung fibroblasts (HFL1; Figure 5C,D) with a log_10_ dilution range of AF38Pep. Over the course of 72 h, TGF-β1 induced the largest mRNA expression of the myofibroblast marker, α-SMA, at the 48 h timepoint in NIH/3T3 cells (Figure 5A). Treatment with the blocking peptide significantly attenuated gene induction at 48 h in all concentrations tested. At the 72 h timepoint, α-SMA mRNA expression was elevated in the untreated control group. However, only AF38Pep treatment at a concentration of 10 µg/mL was sufficient to significantly attenuate the α-SMA mRNA upregulation, which suggested that 0.1–1 µg/mL polypeptide concentrations were not able to effectively inhibit the differentiation response over the 72 h duration of treatment. Cytotoxicity of AF38Pep was assessed to ensure the effects observed were not indicative of NIH/3T3 cell apoptosis (Figure 5B). Indeed, cells maintained a consistent growth pattern in response to TGF-β1 stimulation, and results suggest that treatment with the polypeptide at either of the concentrations did not interfere with cell proliferation nor incur cell death. Similar results were observed when HFL1 cells were assessed for the potential of the blocking polypeptide to attenuate expression of α-SMA (Figure 5C). The upregulation of α-SMA mRNA expression by TGF-β1 was significantly reduced by 0.1–10 µg/mL peptide at 48 h, and only 10 µg/mL was sufficient for inhibition of expression at 72 h. Assessment of AF38Pep toxicity to HFL1 cells (Figure 5D) indicated that the blocking effect was not detrimental to cell numbers.

### 2.4. Evaluation of Polypeptide Binding, Blocking Function and Specificity

**Figure 6:**
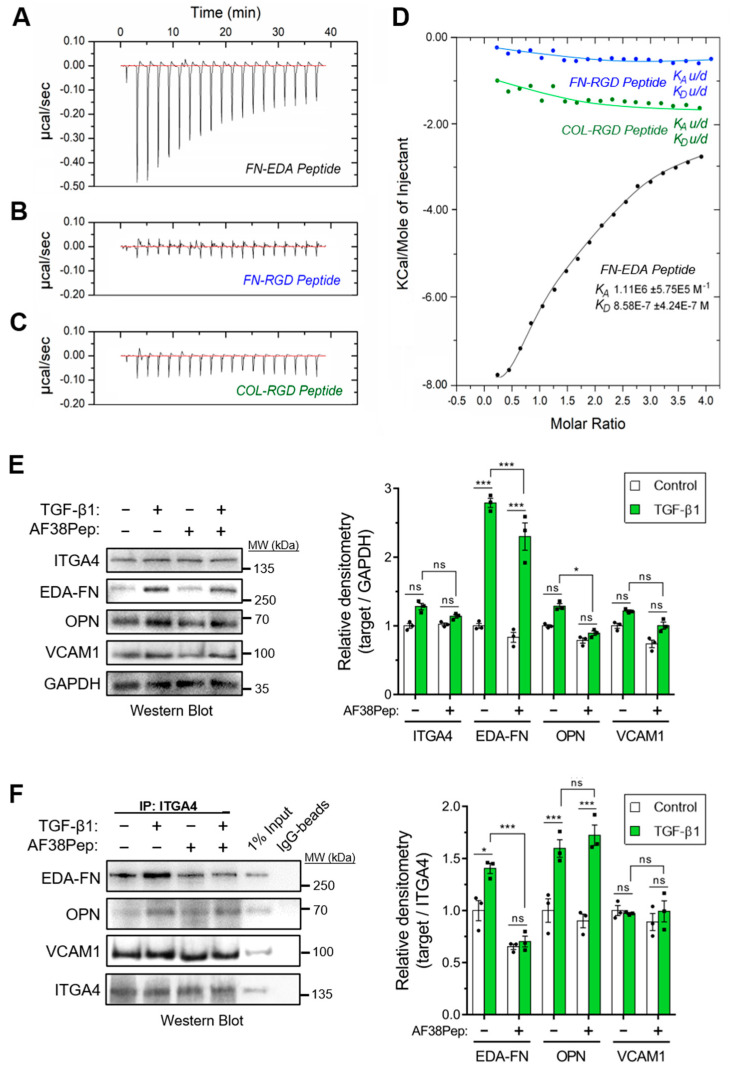
Binding specificity and blocking function of the synthesized blocking polypeptide. The designed blocking peptide was synthesized alongside 3 small test-binding peptides (FN-EDA, FN-RGD, COL-RGD) and were assessed by isothermal calorimetry (ITC), and calorimetric heat traces were used to test whether the blocking peptide has specificity to bind peptides derived from (**A**) the predicted EDA binding ligand (FN-EDA, black), (**B**) the RGD-domain of EDA-FN and FN shown to bind integrin α_4_β_1_/β_7_ at a distal site (FN-RGD, blue), and (**C**) the RGD-domain of collagen I previously shown to bind different integrin subunits (COL-RGD, negative control, green). (**D**) The collated isogram is shown alongside predicted dissociation constants (*K_D_*) and association constants (*K_A_*), calculated using a 1:1 receptor site to ligand site binding mode. (**E**) Western blot analysis of integrin α_4_ protein (ITGA4), EDA-FN, osteopontin (OPN), and vascular cell adhesion protein-1 (VCAM1) total protein following 48 h TGF-β1, AF38Pep or TGF-β1+AF38Pep treatments. GAPDH was used as a loading control. Densitometry quantification is shown alongside. (**F**) Co-IP analysis of ITGA4 binding partners (EDA-FN, OPN, and VCAM1) following 48 h TGF-β1, AF38Pep or TGF-β1+AF38Pep treatments. Data are representative of three independent experiments and displayed as the mean ± S.E. Statistical analysis is shown as *** *p* ≤ 0.001, * *p* ≤ 0.05, and ns = no statistical significance (*p* > 0.05).

The designed AF38Pep blocking polypeptide was synthesized alongside three smaller peptides, each 12 amino acids in length. These smaller peptides were designated as follows: FN-EDA, a peptide sharing amino acid sequence with the C-C′ loop of EDA-FN (TYSSPEDGIHEL); FN-RGD, a peptide derived from the integrin β_1_ and β_3_ subunit-binding RGD motif of fibronectin (YAVTGRGDSPAS); COL-RGD, a peptide derived from the integrin β_1_ subunit-binding RGD motif of collagen I (GPKGDRGDAGPK). Isothermal calorimetry (ITC) was used to determine whether AF38Pep showed interactions with the small peptide FN-EDA (Figure 6A), or unintentional unspecific binding interactions with FN-RGD (Figure 6B) and COL-RGD (Figure 6C). Thermogram plots indicated that there was a detectable interaction between AF38Pep and FN-EDA, but not between AF38Pep and FN-RGD or COL-RGD. The collated isogram plot (Figure 6D) reflected these interactions, as no *K_A_* could be determined for interactions with the peptides derived from RGD domains. A *K_A_* value was calculated for the binding interaction between AF38Pep and FN-EDA, giving evidence of specific binding, affinity and validating the molecular docking predictions used for polypeptide design. These data indicated that the AF38Pep blocking polypeptide was suitable for use for targeting EDA-FN and interfering with its binding to the integrin receptor cleft.

We next assessed the capability of AF38Pep to inhibit EDA-FN interactions with integrin α_4_ protein (ITGA4), and whether interactions with other ligands were also affected. Western blots indicated that the total protein for ITGA4 and its ligand, vascular cell adhesion protein-1 (VCAM1), remained unaffected following TGF-β1 or AF38Pep treatments (Figure 6E). EDA-FN total protein significantly increased following TGF-β1 stimulation compared to the control group. The TGF-β1-driven EDA-FN protein increase was marginally, but significantly, attenuated by AF38Pep treatment. The protein expression of osteopontin (OPN), another ITGA4 ligand, was also significantly attenuated by AF38Pep treatment. To further assess whether AF38Pep interfered with ITGA4–ligand interactions, coimmunoprecipitation (Co-IP) assays were performed using anti-ITGA4 antibodies and Western blot was used to detect interacting proteins (Figure 6F). Co-IP showed that TGF-β1 stimulation increased EDA-FN and OPN binding to ITGA4. The quantity of VCAM1–ITGA4 binding was unaffected by cell treatments and remained at basal levels. AF38Pep specifically inhibited EDA-FN–ITGA4 interactions and did not influence OPN–ITGA4 binding. Taken together, immunoblots demonstrated that although fibroblasts continued to produce EDA-FN protein in response to TGF-β1 stimulation, AF38Pep treatments inhibited EDA-FN binding to the integrin α4 receptor, without affecting the binding of other ligands.

**Figure 7:**
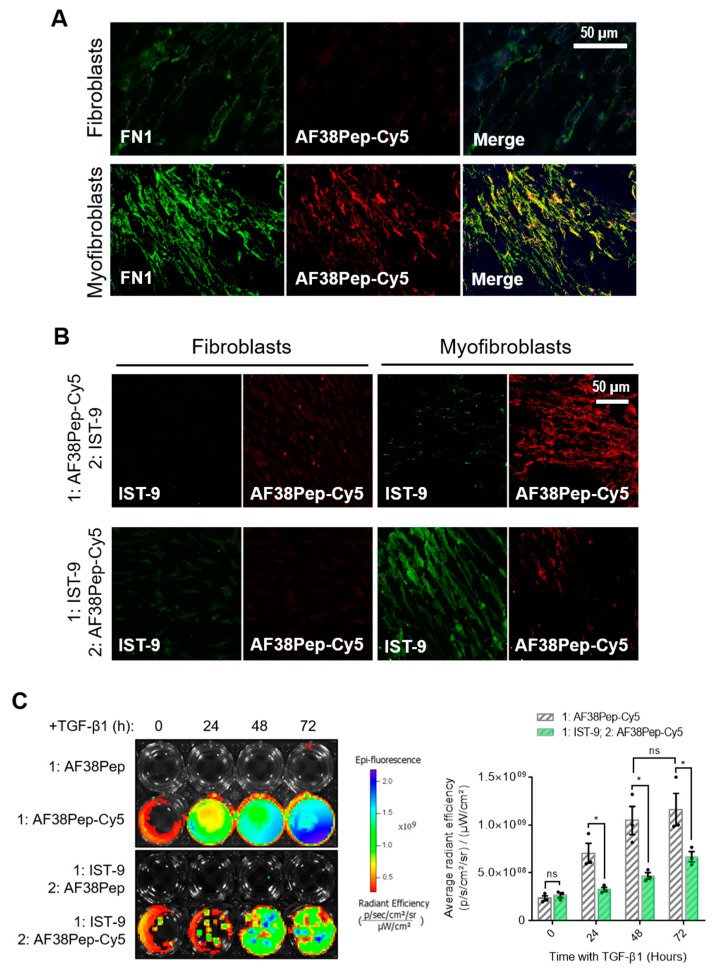
Fibrotic matrix binding characterization of the designed blocking polypeptide. The designed AF38Pep blocking polypeptide was synthesized and conjugated to Cy5 (AF38Pep-Cy5). (**A**) The extracellular matrices (ECMs) of fibroblasts or differentiated myofibroblasts were fixed and co-labelled with fibronectin (FN1) antibody and AF38Pep-Cy5. Original magnification, ×200; scale bar, 50 µm. (**B**) Binding competitiveness was assessed by preincubating fixed matrices with either AF38Pep-Cy5 or IST-9 antibody, followed by incubation with IST-9 or AF38Pep-Cy5, respectively. Original magnification, x200; scale bar, 50 µm. (**C**) Top panel (fibrotic matrix specificity): TGF-β1 was used to stimulate EDA-FN production at the indicated timepoints and 10 µg/mL unlabeled AF38Pep or AF38Pep-Cy5 was added for 1 h before washing and visualization of epifluorescence. Bottom panel (binding specificity): TGF-β1 was used to stimulate EDA-FN production at the indicated timepoints and 10 µg/mL IST-9 antibody was preincubated with cells for 1 h. Cells were washed and then 10 µg/mL unlabeled AF38Pep or AF38Pep-Cy5 was added for 1 h before washing and visualization of epifluorescence. Data are representative of three independent experiments and displayed as the mean ± S.E. Statistical analysis is shown as * *p* ≤ 0.05, and ns = no statistical significance (*p* > 0.05).

To determine AF38Pep affinity for myofibroblast-synthesized ECM, AF38Pep was conjugated to Cy5 dye (AF38Pep-Cy5) and incubated with fixed ECM synthesized by either untreated control fibroblasts or differentiated myofibroblasts. First, localization was assessed by comparing AF38Pep-Cy5 labelling to pan-fibronectin (FN1) immunostaining (Figure 7A). In fibroblasts, some FN1 staining was observable, whereas little to no staining with AF38Pep-Cy5 was observed. In myofibroblasts, FN1 deposition increased and was clearly visualized. AF38Pep-Cy5 labelling also increased in myofibroblasts (red) and merged images showed AF38Pep-Cy5 colocalization with anti-FN1 staining (yellow) was prominent in areas of fibronectin deposition at cell membranes, although regions that were only labelled by FN1 immunostaining were also observed (green). We next compared staining patterns compared to IST-9 (EDA-FN antibody), and whether pretreatments would competitively interfere with labelling (Figure 7B). In fibroblasts, staining with either IST-9 or AF38Pep-Cy5 was minimal, regardless of the order of addition to cells. In myofibroblasts, AF38Pep-Cy5 labelled the upregulated EDA-FN production and pre-treatment blocked IST-9 staining. Similarly, when IST-9 pretreatments were applied, AF38Pep-Cy5 failed to label the elevated EDA-FN production. We further confirmed EDA-FN ECM specificity and competitive binding by incubating AF38Pep-Cy5 with cells treated with TGF-β1 for 24 (early activated fibroblasts), 48 (late activated fibroblasts) and 72 h (differentiated myofibroblasts). The results demonstrated increased binding of AF38Pep to the fibrotic ECM, which correlated with the increasing time of TGF-β1 treatment (Figure 7C). The data showed significant increases in binding at 48 h, compared to 0 h TGF-β1 treatment. Similar results to the immunocytochemistry were observed when pretreatments with IST-9 were made; IST-9 attenuated AF38Pep-Cy5 binding to EDA-FN protein, thereby decreasing the detectable epifluorescence signal over the course of the 72 h TGF-β1 treatment. Taken together, these data suggested that binding sites for AF38Pep-Cy5 were masked by IST-9 pretreatments. AF38Pep also had the capacity to interfere with IST-9 binding, indicating binding specificity of AF38Pep for the EDA-FN protein.

### 2.5. Evaluation of Polypeptide Inhibition of Integrin α_4_β_1_ Signaling

**Figure 8:**
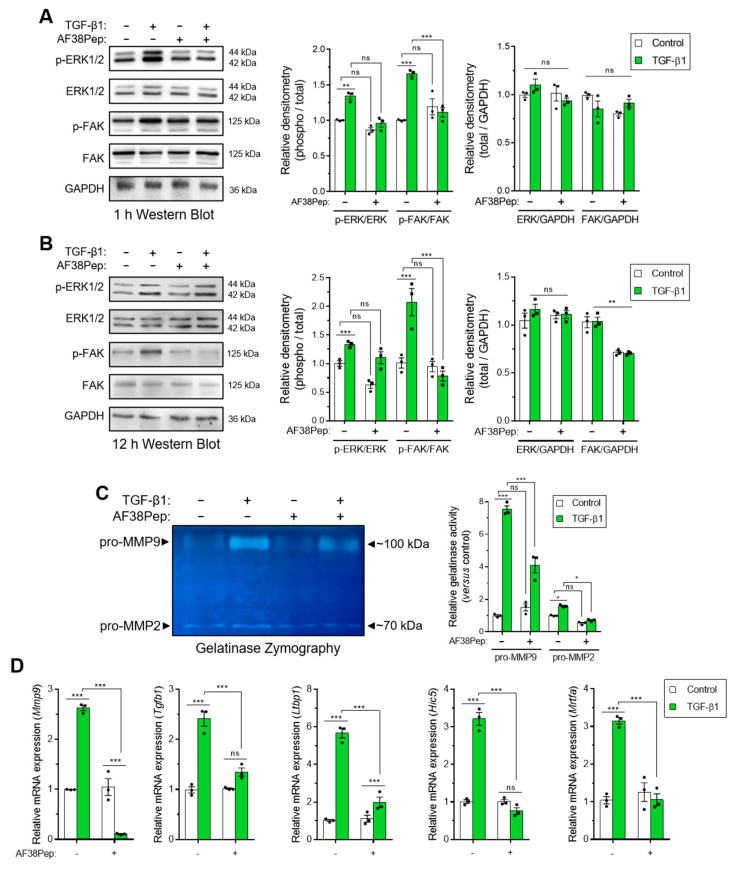
Evaluation of blocking peptide capacity to inhibit integrin α4β1 signaling and downstream profibrotic effects. NIH/3T3 fibroblasts were incubated in the presence or absence of 10 ng/mL TGF-β1 with or without 10 µg/mL AF38Pep for (**A**) 1 h or (**B**) 12 h, before assessment of extracellular-signal-regulated kinases 1 and 2 (ERK1/2) and focal adhesion kinase (FAK) protein expression and phosphorylation. Immunoblots are displayed alongside corresponding densitometry analysis of phospho-protein/total-protein and total-protein/GAPDH loading control. (**C**) Gelatinase zymography indicating pro-matrix metalloproteinase 9 (pro-MMP9) (top band) and pro-MMP2 (bottom band) enzymatic activity following the indicated treatments for 48 h. Shown alongside is the quantification of relative gelatinase activity. (**D**) The mRNA expression for genes associated with integrin α_4_β_1_ activation: *Mmp9*, *Tgfb1*, *Ltbp1*, *Hic5,* and *Mrtfa*, which were assessed by real-time quantitative PCR (qRT-PCR) following 48 h of stimulation by 10 ng/mL TGF-β1. Blots, images, and data are representative of three independent experiments and data are shown as the mean ± S.E. Statistical analysis is shown as *** *p* ≤ 0.001, ** *p* ≤ 0.01, * *p* ≤ 0.05, and ns = no statistical significance (*p* > 0.05).

We next sought to investigate whether events downstream of EDA-FN/integrin α_4_β_1_ binding could be attenuated by our blocking polypeptide. We used Western blotting analysis to examine the signaling proteins, extracellular signal-regulated kinases 1 and 2 (ERK1/2) and focal adhesion kinase (FAK), which were shown to be downstream of integrin β_1_ activation [43,44]. Both phosphorylated forms of ERK1/2 and FAK were significantly upregulated after 1 h of TGF-β1 treatment, and this activation was significantly suppressed when cells were also incubated with AF38Pep (Figure 8A). Early signaling events involving the ERK1/2 pathway have been implicated in fibronectin-mediated integrin signaling [45]. Indeed, ERK1/2 activation was not prevented at 12 h, when cells were simultaneously incubated with AF38Pep and TGF-β1 (Figure 8B). This result may be explained by epidermal growth factor receptor (EGFR) and TGFβRI/II pathway-dependent activation of ERK1/2, which induced phosphorylation in a biphasic and time-dependent manner following the stimulation of fibroblasts with TGF-β1 [46]. Alternatively, the delayed ERK1/2 signaling may have been in response to FN type III, OPN or VCAM1 binding to their integrin receptor sites at distal locations [47]. However, phosphorylation of FAK protein showed significantly less activation, indicating the prolonged inhibition of the integrin/FAK signaling pathway could be achieved by the AF38Pep blocking polypeptide. Interestingly, total protein levels of FAK were also marginally reduced by the blocking polypeptide at 12 h, suggesting inhibitory actions on the FAK pathway were two-fold; the reduction in activation and reduction in total protein synthesis. Use of the IST-9 antibody resulted in similar signaling inhibition of FAK at 12 h post-treatment (Figure S1). However, IST-9 prevention of ERK1/2 phosphorylation at the 12 h timepoint suggested the blockade of additional non-EDA-FN-specific ITGA4 activation may have been implicated [47]. Matrix metalloproteinase (MMP) gelatinase release and activity has been associated with fibronectin-dependent activation of integrin α_4_β_1_ and other β_1_-containing integrins [48,49,50]. We used gelatin zymography to assess gelatinase activity in conditioned medium taken from peptide-treated cells (Figure 8C). The results demonstrated a significant upregulation of both pro-MMP9 and pro-MMP2 gelatinase activity following incubation of fibroblasts with TGF-β1 for 48 h (untreated lane 1 versus TGF-β1-treated lane 2). Both pro-MMPs exhibited suppressed activity levels when TGF-β1-treated fibroblasts were also incubated in the presence of AF38Pep (TGF-β1-treated lane 2 versus TGF-β1+AF38Pep-treated lane 4). Genes indicative of integrin α_4_β_1_ activation, downstream of TGF-β1 stimulation, were also attenuated by AF38Pep treatment (Figure 8D). These included the mRNA expression for MMP9, TGF-β1, latent-TGF-β-binding protein 1 (LTBP1), TGF-β1-induced transcript 1 (HIC5), and myocardin-related transcription factor A (MRTFA), which gave strong evidence that the polypeptide had successfully blocked the interaction between EDA-FN and integrin α_4_β_1,_ and thus prevented the subsequent upregulation of these integrin activation-dependent genes. Overall, the data suggested that AF38Pep interfered with EDA-FN/integrin binding, attenuated FAK-dependent signaling, decreased MMP2 and MMP9 production and activation, and prevented the upregulation of integrin activated gene expression.

### 2.6. Evaluation of Polypeptide Inhibition of Profibrotic Cell Activity

**Figure 9:**
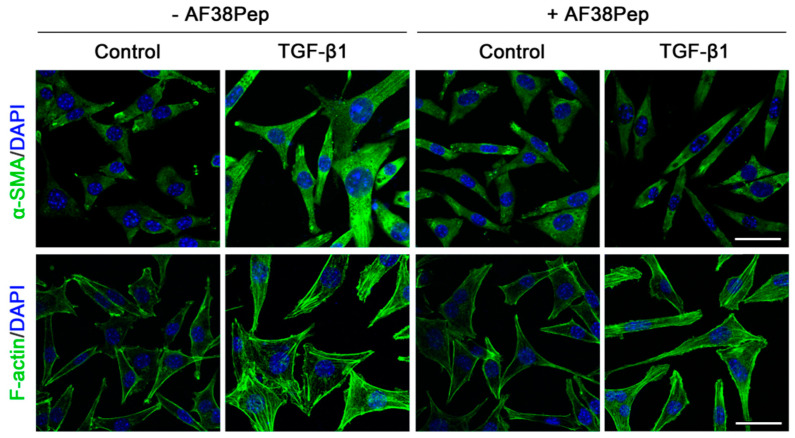
Assessment of blocking peptide effect on myofibroblast phenotype acquisition. NIH/3T3 fibroblasts were incubated in the presence or absence of 10 ng/mL TGF-β1 with or without 10 µg/mL of peptide for 48 h; immunocytochemistry was used to visualize the marker of myofibroblast differentiation (α-SMA, green; DAPI, blue; top row; original magnification, x200; scale bar, 25 µm) and an indicator of fibroblast activation to a proliferative, migratory and synthetic phenotype (F-actin, green; DAPI, blue; bottom row; original magnification, x200; scale bar, 25 µm). Images are representative of three independent experiments.

**Figure 10:**
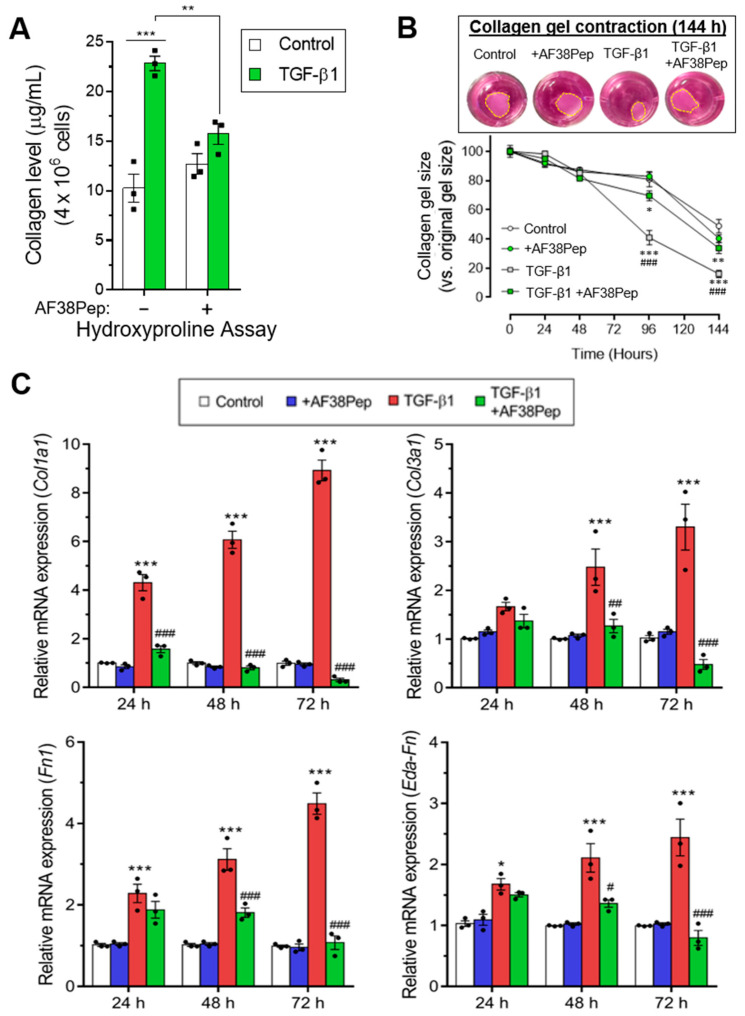
Assessment of blocking peptide effect on profibrotic activity and integrin α4β1-induced gene expression. (**A**) The quantification of total collagen production by NIH/3T3 cells as calculated from hydroxyproline assays after 48 h of treatment time. (**B**) Collagen gel contraction assay over the course of 144 h in the absence or presence of the small blocking peptide and 10 ng/mL TGF-β1. The contracted collagen gel images are shown at the 144 h timepoint. Yellow dashed lines show perimeters of contracted gels. The mRNA expression for (**C**) fibrosis-associated genes: *Col1a1*, *Col3a1*, *Fn1,* and *Eda-Fn1* were assessed by qRT-PCR at 24, 48 and 72 h. Data are representative of three independent experiments and shown as the mean ± S.E. Statistical analysis is shown as *** *p* ≤ 0.001, ** *p* ≤ 0.01, and ns = no statistical significance (*p* > 0.05). #, ##, ###—statistically significant difference in comparison to TGF-β-only stimulated cells.

Immunocytochemistry for α-SMA stress fiber formation confirmed the results shown by mRNA analyses (Figure 9, top row). Protein expression of α-SMA was inhibited when TGF-β1-treated NIH/3T3 dermal fibroblasts also received 10 µg/mL AF38Pep treatment, suggesting a lack of myofibroblast phenotypes. Activated fibroblasts reassemble their cytoskeletons to form long F-actin fibers that extend along the length of the cells [51,52]. Thus, we sought to assess whether this process was prevented in AF38Pep-treated cells (Figure 9, bottom row). Our results indicated that although cells failed to acquire enlarged morphologies, cellular actin reassembly into F-actin fibers still occurred in the presence of AF38Pep treatment. This observation suggested that myofibroblast maturation could be attenuated but fibroblasts still progressed to an activated state—often associated with a synthetic fibroblast phenotype (or proto-myofibroblast) with elevated proliferation and migration [51,53]. EDA-FN binding to integrins α_4_β_1_ and α_4_β_7_ was shown to be causative of mechano-transduction and resultant collagen-rich ECM production [32,38]. Total collagen synthesis, as determined by hydroxyproline assay (Figure 10A), demonstrated reduced total collagen synthesis to approximately 60% of that observed in cells that only received TGF-β1 treatment, a similar level to collagen production by untreated control fibroblasts. Cell contractility was attenuated by AF38Pep treatment (Figure 10B), which corroborated the inhibition of α-SMA incorporation into the cytoskeleton. Cotreatment of AF38Pep alongside TGF-β1 stimulation significantly delayed collagen gel contraction by fibroblasts at 96 h and maintained a significant difference in contraction at 144 h, when compared to TGF-β1 stimulation alone. Subsequent analyses of the expression of genes associated with profibrotic myofibroblast phenotypes and indicative of fibrogenesis (Figure 10C) revealed that the upregulated mRNA expressions of collagen types I and III, fibronectin and the EDA-FN splice variant by TGF-β1 were all inhibited when AF38Pep was also incubated with the fibroblasts for 72 h. Expression of fibrotic markers in TGF-β1- and AF38Pep-treated cells marginally increased at 24 and 48 h. However, these failed to reach expression levels exhibited by TGF-β1-only stimulated cells, with significant attenuation being shown across all assessed genes by 48 h. Overall, the data suggest that AF38Pep interference with EDA-FN/integrin binding prevented the enrichment of collagens and the synthesis of a profibrotic ECM, whilst enabling fibroblasts to enter an activated state, but not permitting the progression to mature myofibroblast phenotypes.

## 3. Discussion

The persistent presence of EDA-FN in tissues is a hallmark of fibrosis, synthesized by activated fibroblasts and myofibroblast cells, which continues to drive the development of progressive fibrotic diseases. In this investigation, we used in silico docking simulations to design a polypeptide, based on the integrin α_4_β_1_ receptor binding site for the EDA-FN C-C′ loop. The polypeptide that performed the best in molecular docking simulations was selected for synthesis and subsequent in vitro assessment. We showed that the EDA-FN binding and blocking polypeptide, that we named AF38Pep, was specific for myofibroblast-synthesized matrices which contain EDA-FN, only associated with the EDA-FN C-C′ loop and showed no associations with fibronectin or collagen RGD domains. We also showed that AF38Pep specifically interfered with EDA-FN–integrin α_4_β_1_ interactions, attenuated subsequent ERK1/2 and FAK signaling, and prevented downstream mRNA expression of associated genes. AF38Pep also attenuated collagen synthesis and prevented activated fibroblast differentiation into mature myofibroblasts. The data shown here provide promising evidence for this first generation of EDA-FN small blocking polypeptides that could be implemented in a broad range of pathological conditions related to EDA-FN activity.

The EDGIHEL motif within the EDA-FN C-C′ region can bind to fibroblast-expressed integrins, including α_4_β_1_ and α_4_β_7_, and the α_4_ subunit was suggested to be an essential EDA-binding component [30,44,54]. From the data shown here, in addition to α_4_ motifs, there was clearly strong affinity for the β_1_ subunit. When performing in silico assessment of the designed small blocking polypeptides, the polypeptides that consisted of motifs found within the β_1_ subunit produced superior binding affinity scores, compared to those that used the β_7_ subunit motifs. This suggested that the EDA-FN C-C′ loop may have a higher binding affinity for integrin receptor dimers that have β_1_ subunits. In combination with previous research, it could be speculated that α_4_β_1_ produces the highest binding affinity, whereas integrin α_4_β_7_ may have less affinity due to suboptimal subunit dimer pairings for EDA-FN binding.

The small blocking polypeptide designed as part of this investigation was optimized by mimicking the α_4_β_1_ receptor site. Despite utilizing integrin α_4_β_1_ binding motifs, AF38Pep was designed to bind to the EDA-FN C-C′ loop. Thus, in addition to blocking interactions with α_4_β_1_, interactions with other receptors that specifically interact with the EDA-FN C-C′ loop may also be prevented by AF38Pep treatment. This broadens the implications of the polypeptide in blocking multiple cell-signaling events that rely on EDA-FN C-C′ loop binding and activation of receptors and warrants investigation in future studies. Integrin α_4_β_7_ was shown to mediate EDA-FN-induced expression of α-SMA containing stress fibers, collagen synthesis, and cellular contractility in lung fibroblasts [32]. Integrin α_4_β_1_ activation by EDA-FN promoted an increase in the assembly of actin and the activation of myosin light chain (MLC), promoted cytoskeletal stress fiber formation, increased fibronectin expression and fibrillogenesis, and led to matrix stiffening but not α-SMA incorporation into stress fibers [38]. It is not known whether one of these integrin activation events precedes the other, but matrix stiffening by the upregulated production of EDA-FN is a prerequisite for myofibroblast stress fiber formation, suggesting α_4_β_1_ signaling events leading to matrix deposition may precede α_4_β_7_ signaling leading to stress fiber formation. Regardless of the order of integrin signaling events, AF38Pep had the capacity to attenuate both matrix deposition and α-SMA stress fiber formation. These outcomes suggested that AF38Pep was capable of global inhibition of EDA-FN C′C′ loop interactions with cell surface integrins. Although the epithelial-expressed integrin α_9_β_1_ has suggested roles in driving EMT, specific mechanisms of regulation of the transitional events remains unknown [31,54]. EMT in lung and colorectal cancer is accompanied by induction of the expression of mesenchymal markers, including α-SMA [54] and vimentin [21], and is thought to be a contributing source of myofibroblasts within fibrotic tissues [55]. Therefore, we speculate that EDA-FN interactions with integrin α_9_β_1_ on epithelial cells may be prevented by AF38Pep treatment. Thus, in addition to treatment of profibrotic disease cell types such as those isolated from keloids and hypertrophic scars [24], the EMT process will also be a focus for future investigations into the broader implications of our designed small blocking polypeptide.

In addition to integrins, a recent study reported EDA-FN-dependent signaling through toll-like receptor 4 (TLR4) drove fibrosis in scleroderma [25]. EDA-FN is a ligand for TLR4 and EDA-FN/TLR4 binding resulted in the upregulation of proinflammatory cytokines [56,57]. It was also shown that EDA-FN activated TLR4 expressed on human dermal fibroblasts, induced interleukin-8 (IL-8) and tumor necrosis factor-α (TNFα) expression [24], and promoted TLR4-dependent synthesis of collagen, which implicated EDA-FN/TLR4 in both proinflammatory and profibrotic cell responses [6,25,57]. Whether AF38Pep could also prevent EDA-FN recognition by TLR4 is not known. TLR4 is an inflammatory signaling regulator that warrants investigation, especially in designing therapies against the vicious cycle of chronic inflammation and fibrosis-related pathologies suggested to arise in response to EDA-FN [24,57]. Stromal fibroblast or myofibroblast activation, metastasis, and vasculogenesis have been documented as being initiated by the production of EDA-FN [20,21,58]. Therefore, in addition to providing a potential means to block the development of stromal myofibroblasts, AF38Pep may have utility as a targeting or theranostic labelling method for fibrosis or tumors, especially given the rarity of EDA-FN in adult tissues. These various avenues of investigation will form part of future research exploring additional usage of AF38Pep and its broader activities.

Our observations suggested that prolonged FAK activation was attenuated by specific interference with integrin EDA-FN/integrin α_4_β_1_ interactions. Previous reports have suggested that ERK1/2 signaling was mediated by integrin α_4_β_7_ [32]. We observed that early ERK1/2 signaling at 1 h post-TGF-β1 stimulation was diminished by treatment with AF38Pep, whereas ERK1/2 signaling at 12 h was not. This may be plausibly explained by previous research that showed that signaling through ERK1/2 is a temporally controlled process involving multiple receptors [46], or may involve participation from other integrin ligands, OPN and VCAM1 [47]. It remains unclear whether TGF-β1 stimulated ERK1/2 activation can be further regulated by integrin signaling in a time sensitive or cyclic manner. In this study, our results suggested that early ERK1/2 signaling and prolonged FAK activation were dependent on the EDA-FN C-C′ loop binding to the integrin α_4_β_1_ or integrin α_4_β_7_ receptor cleft.

Removal of EDA-FN from the genome or proteome has been shown to be detrimental to tissue remodeling during wound healing [18]. Olsen et al. showed that removal of EDA-FN from hepatic stellate cells and portal fibroblasts failed to prevent myofibroblast transdifferentiation in culture or in liver fibrosis models [59]. This unexpected observation was explained in a study by Kawelke et al., wherein genetic deletion of FN increased fibroblast numbers and TGF-β1 production, which led to increased fibrosis [60]. This was later confirmed in an investigation by Iwasaki et al. which demonstrated the accumulation of highly disorganized collagenous ECM networks in FN-null liver injury [61]. The unanticipated upregulation in ECM stiffness from increased collagen I-rich matrix deposition and dysregulation of TGF-β1 contributed to a more aggressive fibrotic phenotype. Thus, there is clearly a regulatory mechanism of balance in place between FN and collagen synthesis and arrangement, which is important in tuning matrix stiffness and regulating myofibroblast. Therefore, a highly specific blockade of EDA-FN C-C′ loop interaction with integrin α_4_β_1_ and the attenuation of downstream intracellular signaling pathways are required for myofibroblast differentiation; inhibiting EDA-FN/integrin α_4_β_1_-mediated myofibroblast formation without unnecessarily interfering with additional collagen and TGF-β1 regulating functions of the EDA-FN and other FN variants is a desirable approach. In simpler terms, the EDA-FN macromolecule can remain within the matrix to convey its other important functions, whilst the small blocking peptide can attenuate EDA-FN C-C′ loop/integrin α_4_/β_1_-dependent signaling.

The blocking polypeptide shown here could serve to prevent initial EDA-FN-dependent myofibroblast transformation and collagen upregulation that would otherwise contribute to the persistence of myofibroblasts in tissues. However, the synthesis and deposition of fibrillar collagen I in progressive fibrosis appears to take over the role of the predominant source of ECM mechanical stress and becomes the principal driver of the myofibroblast phenotype [62]. Therefore, treatment of established fibrosis using EDA-FN blocking antibodies or small blocking polypeptides may be ineffective in reversing the process. However, by utilizing AF38Pep as a targeting method, bioactive factors that have been suggested to reverse the myofibroblast phenotype, e.g., BMP7 [52], HGF variants [63], or CD44 variants [52,64] etc., could be conjugated to increase the efficacy of the treatment, preventing new myofibroblast formation whilst reversing the established phenotypes.

In conclusion, the small blocking polypeptide, AF38Pep, was modeled from the α_4_β_1_ receptor binding site for EDA-FN. AF38Pep was subsequently synthesized for use in in vitro assessment of antifibrotic activity and exhibited a prominent antifibrotic effect; reduced TGF-β1 stimulated fibronectin and collagen production; prevented integrin signaling through FAK; attenuated downstream release and activation of MMPs, and integrin signaling-associated gene expression, and cumulated in the inhibition of mature myofibroblast formation. The blocking polypeptide reported here has potentially broad applications as an antifibrotic treatment to restrict myofibroblast transdifferentiation, progressive fibrosis, and related diseases.

## 4. Materials and Methods

### 4.1. Materials

Cell culture reagents and plasticware were purchased from Sigma-Aldrich (Poole, Dorset, UK), unless otherwise stated. Custom peptides were synthesized by ChinaPeptides (Shanghai, China). Cells used for experiments were human lung fibroblasts (HFL1; CCL-153, ATCC, Manassas, VA, USA), kindly provided by Professor Wen Ning (Nankai University, Tianjin, China), and mouse dermal fibroblasts (NIH/3T3; CRL-1658, ATCC). TGF-β1 was purchased from R&D Systems (Shanghai, China). All antibodies were purchased from Abcam (Cambridge, UK).

### 4.2. Generation of Integrin α4β1 Protein Model

Integrin β_1_ (ITB1_human) has 42.112% amino acid sequence homology with integrin β_7_ (ITB7_human). Thus, integrin α_4_β_7_ was used as a template upon which the integrin β_1_ subunit was mapped onto to create a working integrin α_4_β_1_ model. Chimera v1.13.1 (UCSF, San Francisco, CA, USA) and MODELLER v9.21 (UCSF) (Figure S2) software suites were used to create the integrin α_4_β_1_ model. Briefly, the resolved crystal structures of the α_4_β_7_ head domains (3V4V; RCSB PDB) and the full ITB7_human sequence were used as references for the structural mapping and overlaying of ITB1_human onto the crystal structure of 3V4V, and to simulate association with the integrin α_4_ subunit whilst in active conformation. The structural orientations of the integrin β_1_ subunits were compared between the newly generated α_4_β_1_ model and the resolved crystal structure of integrin α_5_β_1_ (4WK2; RCSB PDB); similarities between sequence and head domain alignments were used to validate the mapping of the integrins. As a further validation control for predicted binding sites, the integrin α_4_ (ITA4_human) was mapped onto the 4WK2 resolved model, using the integrin α_5_ (ITA5_human) sequence as a reference template. Receptor model quality was assessed and ensured through examination using PROCHECK (EMBL-EBI, Cambridge, UK) (Figure S3) and Verify3D (UCLA-DOE Institute, Los Angeles, CA, USA) (Figure S4).

### 4.3. Molecular Docking and Generation of Blocking Peptide Models

The EDA-FN ligand (YSSPEDGHIEL) and its flanking amino acid sequence were isolated from the NMR structure (1J8K; RCSB PDB) and the structure was minimized with AMBER protocols (AmberTools18, UCSF). Chimera v1.13.1 (UCSF) software suite was used to create the integrin α_4_β_1_ (generated in-house) and integrin α_4_β_7_ (from 3V4V) receptor docking compatible models, and the EDA-FN peptide docking ligand model. Molecular docking simulations were performed using AutoDock Tools and AutoDock Vina (Scripps Research, San Diego, CA, USA). Briefly, a total of five grids were selected to examine the entirety of the integrin receptor surfaces. Each grid was analyzed individually for simulated molecular docking of the EDA-FN peptide ligand with the AutoDock settings to add polar hydrogens and to perform molecular docking at an exhaustiveness of 8. The top 7 binding modes from each analysis were visualized in PyMOL v2.2.3 (Schrödinger K.K., Tokyo, Japan) and colorized based on affinity score. The electrostatic potentials of integrin receptor surfaces, hydrogen bond predictions, and polar contacts between receptor and ligand were assessed using PyMOL v2.2.3 (Schrödinger K.K.) built-in scripts. The PEP-SiteFinder tool (RPBS, Paris, France [65]) was used to strengthen predictions of candidate amino acid binding regions. To generate a series of blocking peptides, the amino acids of the integrin receptors that had polar contacts that within a range of ≤3 Å with the ligand were listed and the amino acids predicted to form hydrogen bond donors were noted. A series of 10 peptides modeled from the integrin receptor site were generated based on native receptor sequence and the key predicted interacting amino acid regions. Peptides were manually constructed, and initial folding simulations were run in PyMOL v2.2.3 (Schrödinger K.K.). Next, peptides were exported to Chimera v1.13.1 (UCSF) and minimalized by AMBER (UCSF) protocols. Peptides were further assessed and narrowed down to 5 choices based upon AMBER (UCSF), PROCHECK (EMBL-EBI), and RaptorX Contact Prediction (University Chicago, Chicago, IL, USA) scores. The 5 best peptide derivatives were uploaded to PEP-FOLD3 (RPBS [66,67,68]) and each were assessed for the 5 best folding conformations, which in turn were then assessed for the 5 best binding conformations with the highest docking affinity to the EDA-FN peptide (YSSPEDGHIEL) using AutoDock Vina (Scripps Research). The peptide that had the overall highest binding score was chosen for synthesis and further experimentation.

### 4.4. Cell Culture

HFL1 cells were cultured in Kaighn’s modified Ham’s F-12 (F-12K) medium (Sigma-Aldrich) containing NaHCO_3_ and HEPES buffered to pH 7. Cells were grown to confluence in T75 culture flasks in the presence of 10% fetal bovine serum (FBS; Gibco, Thermo Fisher Scientific, Waltham, MA, USA). HFL1 cells were subcultured using 0.05% trypsin at a ratio of 1:3. NIH/3T3 cells were cultured in Dulbecco’s Modified Eagle Medium (DMEM; HyClone, Thermo Fisher Scientific) containing 100 U/mL penicillin and 100 μg/mL streptomycin. Cells were grown to confluence in T75 culture flasks in the presence of 10% FBS. NIH/3T3 cells were subcultured using 0.05% trypsin/EDTA at a ratio of 1:3. All cells were maintained at 37 °C and 5% CO_2_, and growth medium was replenished every 3 days until experimentation. Cells were growth-arrested in serum-free medium for 48 h before experiments, and all experiments were performed under serum-free conditions using cells between passages 3 and 8.

### 4.5. Cell Viability Assay

Cells were seeded into 12-well culture plates at a density of 2.5 × 10^4^ and when cultures reached 50% confluence, the culture medium was replaced with fresh serum-free F12K or serum-free DMEM/F12 for 48 h (growth arrest). Cells were treated with the indicated concentration of peptide for 48 h in the presence or absence of 10 ng/mL TGF-β1. Cell Counting Kit-8 (CCK-8; Beyotime, Haimen, China) assays were performed as follows: 10% of CCK-8 reagent was added to the target wells (90% F12K or 90% DMEM). CCK-8 utilizes water-soluble tetrazolium salt-SST-8 (WST-8), which is reduced by dehydrogenases of cells, to give soluble orange colored formazan. The amount of the formazan dye generated by dehydrogenase was directly proportional to the number of living cells on plates during the 4 h incubation period. Medium was aliquoted into wells of a 96-well plate. Absorbance was measured at 450 nm wavelength using a Bio-Rad optical plate reader.

### 4.6. Real-Time Quantitative Polymerase Chain Reaction (qRT-PCR)

Reverse transcription (RT) and real-time quantitative PCR (qRT-PCR) were used to assess *ACTA2*/*Acta2*, *Col1a1*, *Col3a1*, *Fn1*, *Eda-Fn1*, *Mmp9*, *Tgfb1*, *Ltbp1*, *Hic5,* and *Mrtfa* mRNA expressions. Custom PCR primers were designed in-house (Table 2) and then synthesized by BGI (Beijing, China). Cells were grown in 6-well culture plates and washed with phosphate buffered saline (PBS) before lysis with TRIzon Reagent (CWBio, Beijing, China) and total RNA purification according to the manufacturer’s protocol. Reverse transcription was carried using the TransScript First-Strand cDNA Synthesis SuperMix kit according to the manufacturer’s protocol (TransGen, Beijing, China), using the random primer method for the initiation of cDNA synthesis. Nuclease-free H_2_O replaced the RNA sample to serve as a negative control for the reverse transcription reaction. For qRT-PCR, 10 µL 2× TransStart Top Green qPCR SuperMix was added to each well of a 96-well PCR plate containing 0.4 µL forward primer, 0.4 µL reverse primer, 500 ng RNA, and nuclease-free water to a total volume of 20 µL. The qRT-PCR reaction was completed using a CFX96 Real-Time PCR Detection System (Bio-Rad Laboratories, Hercules, CA, USA). Amplification used the following parameters: 94 °C for 30 s, followed by 40 cycles of 94 °C for 5 s, 55 °C for 15 s, 72 °C for 10 s, and then a dissociation stage (melt-curve). Simultaneous qRT-PCR reactions were performed for *GAPDH*/*Gapdh* as a standard reference gene and using nuclease-free water in the place of cDNA as a negative control.

### 4.7. Isothermal Calorimetry of Peptide Pairs

Custom designed peptides were resuspended in sterile-filtered HEPES buffered saline (140 mM NaCl, 1.5 mM Na_2_HPO_4_, 50 mM HEPES; pH 7). The larger EDA-FN binding peptide was diluted to 15 µM, degassed and added to the calorimetry chamber well. Three smaller test peptides (fibronectin EDA-peptide, FN-EDA; fibronectin RGD-peptide, FN-RGD and collagen I RGD-peptide, COL-RGD) were diluted to 300 µM, degassed and each was separately loaded into the calorimetry injection needle. The chamber well temperature was kept consistent at 25 °C. Blank buffer was used to replace either the well peptide or the injection needle peptide to account for background heat generation. Isothermal titration calorimetry was performed using a MicroCal iTC200 (Malvern Panalytical, Malvern, UK) and dissociation constant (*K_D_*) and association constant (*K_A_*) were calculated using the one set of sites model fitting (with number of binding sites assumed to be 1) in MicroCal Origin Software v8.5 (Malvern Panalytical).

### 4.8. Labelling of Peptide with Cy5 and Epifluorescence Assay

A solution containing 1:1 molar ratio of AF38Pep to non-sulphated Cy5-NHS-ester (APExBIO, Houston, TX, USA) was incubated overnight in RT, shielded from light and under constant mixing. The following day, the solution was passed through a PD-10 Sephadex G-25 M gel filtration column (GE Healthcare, Chicago, IL, USA) into PBS. The first 3 fractions (highest molecular weight) were collected, dialyzed (membrane MWCO 1 kDa) against ddH_2_O overnight, and concentrated by vacuum centrifuge at 3000 rpm at 60 °C for 3 h. Fibroblasts were grown to 70% confluence in 12-well tissue culture plates, before growth arrest in serum-free DMEM for 48 h. Cells were then treated with 10 ng/mL TGF-β1 for 0, 24, 48 and 72 h. The conjugated AF38Pep-Cy5 was added to the plate wells at 10 µg/mL and was incubated for 2 h. The culture medium was removed, and cells were washed three times with PBS before replenishment with fresh serum-free DMEM. Any AF38Pep-Cy5 that remained bound to EDA-FN was assessed using an IVIS imaging system (Caliper Life Sciences, PerkinElmer, Hopkinton, MA, USA) set to detect Cy5.5 epifluorescence after excitation at 640 nm. Epifluorescence was quantified using Living Image v4.2 analysis software (Caliper Life Sciences).

### 4.9. Western Blot

Total protein was extracted from cells in RIPA lysis buffer containing 1% protease inhibitor cocktail, 1% phenylmethylsulfonyl fluoride, and 1% sodium orthovanadate (Santa Cruz Biotechnology, Santa Cruz, CA, USA). Protein was quantified by Micro BCA Protein Assay Kit (Thermo Fisher Scientific) before 7.5% SDS-PAGE and transfer to nitrocellulose. Membranes were blocked with 5% bovine serum albumin (BSA; Sigma-Aldrich)/0.5% Tween-20 (Sigma-Aldrich)/PBS for 1 h, RT, followed by incubation with primary antibodies diluted in 1% BSA/0.1% Tween-20/PBS, overnight at 4 °C. Primary antibodies were: anti-phosphoY204/Y187 ERK1/2 (1:1000); anti-ERK1/2 (1:1000); anti-phosphoY861 FAK (1:1000); anti-FAK (1:2000). Immunoblotting for anti-GAPDH (1:2500) was used as a protein loading control. Following wash steps, membranes were incubated in goat anti-rabbit IgG H&L secondary antibody (horseradish peroxidase, HRP conjugate, 1:10,000) (Abcam; 1:5000 dilution, 1% BSA/0.1% Tween-20/PBS). Detection was performed using Immobilon reagent (Merck Millipore, Burlington, MA, USA) and imaged using a Tanon 5500 Chemiluminescent Imaging System (Tanon, Shanghai, China).

### 4.10. Coimmunoprecipitation (Co-IP)

A volume of 25 µg total protein from cell lysate in nondenaturing lysis buffer containing protease inhibitors was immunoprecipitated (IP) using anti-ITGA4 antibodies linked to protein A/G sepharose beads (Abcam). IP was completed according to the manufacturer’s protocol (ab206996, Abcam). The protein/Ab-bead complexes were centrifuged for 1 min at 2000× *g*. Supernatants were discarded, and beads were washed with 0.1% BSA in PBS. Elution was performed by boiling beads in protein loading buffer for 5 min. Western blotting analysis was performed to determine coeluted proteins.

### 4.11. Immunocytochemistry

Cells were grown to 40% confluence in 8-well glass chamber slides (Nunc, Thermo Fisher Scientific), prior to growth arrest and treatment. Cells were washed with PBS before fixation in 4% paraformaldehyde for 10 min at room temperature. Next, cells were permeabilized with 0.1% (*v*/*v*) Triton X-100 in PBS for 10 min at room temperature. For visualization of ECM, cells were fixed with 100% ice-cold methanol for 8 min at −20 °C. Slides were washed with PBS and blocked with 1% goat serum (TBD Science, Tianjin, China) for 30 min before washing with 0.1% (*w*/*v*) BSA in PBS. Subsequently, slides were incubated with monoclonal mouse anti-α-SMA antibody (Sigma-Aldrich, 1:50) diluted in 0.1% BSA-PBS, overnight at 4 °C. After further wash steps, slides were incubated with goat anti-mouse IgG CF-488 secondary antibody (Abcam, 1:1000) in 0.1% BSA-PBS for 1 h at room temperature and under darkness. For F-actin visualization, after fixation and permeabilization; cells were incubated with phalloidin conjugated to FITC (Sigma-Aldrich) in 0.1% BSA-PBS, overnight at 4 °C. Cells were then mounted in DAPI Fluoromount-G (Thermo Fisher Scientific) and imaged using a Zeiss Axio Imager Z1 UV fluorescence microscope (Zeiss, Jena, Germany) and a Leica TCS SP5 laser scanning confocal microscope (Leica Biosystems, Baden-Württemberg, Germany).

### 4.12. Gelatinase Zymography

Conditioned media were collected from cells incubated with serum-free medium (SFM) alone, SFM containing 10 ng/mL TGF-β1, SFM containing 10 µg/mL AF38Pep, or SFM containing both 10 ng/mL TGF-β1 and 10 µg/mL AF38Pep. Conditioned medium was concentrated 10× by filtrating spin columns (Amicon Ultra-4 10 kDa MWCO, Merck Millipore) and protein was quantified by Micro BCA Protein Assay Kit (Thermo Fisher Scientific). A 1.5 mm thick 10% nonreducing acrylamide gel containing gelatin was prepared and 10 µg of sample in 5 × nonreducing buffer was loaded into the gel lanes. The gel was run at 100 V until the protein entered the stacking gel layer, then the voltage was increased to 150 V until the marker achieved resolved band separation. The gels were then washed twice for 30 min using wash buffer to remove SDS and renature the enzymes. Gels were further rinsed for 5–10 min in incubation buffer at 37 °C with agitation to activate the gelatinase reaction. Fresh incubation buffer was added, and the gels were incubated overnight at 37 °C. Gels were stained with staining solution containing Coomassie blue for between 30 min to 1 h, before rinsing with ddH_2_O and subsequent incubation with de-stain solution until bands could be observed. The gels were transferred to 2% acetic acid and rehydrated overnight for imaging by scanner.

### 4.13. Hydroxyproline Assay

Approximately 4 × 10^6^ of TGF-β1- and/or AF38Pep-treated NIH/3T3 fibroblast cells were harvested and homogenized in 1:1 conditioned medium to HCl (10N), before incubation at 120 °C for 24 h or until complete sample hydrolyzation. Cell-associated and soluble collagen contents were assessed using the Chondrex Hydroxyproline Assay kit (Chondrex, Redmond, WA, USA) according to the manufacturer’s protocol. In brief, 10 µL of sample, standard or blank control solution was added in duplicate to a 96-well clear-bottomed absorbance plate. Next, 100 µL of 1× chloramine-T solution was added to the wells and incubated at room temperature (RT) for 20 min. After incubation, 100 µL 1× DMAB solution was added to the wells and incubated at 60 °C for 30 min, with mild agitation at 5 min intervals. The plate O.D. values were read at 540 nm and sample values were compared to the values of the standard curve. The total collagen content was determined using the following equation:

Collagen level (µg/mL) = Hydroxyproline level (µg/mL) × (100/13.5)

### 4.14. Collagen Gel Contraction Assay

Approximately 2 × 10^4^/mL NIH/3T3 fibroblasts were added to a solution containing 3 mg/mL rat tail collagen type I (BD Biosciences, San Diego, CA, USA), 260 mM NaHCO_3_, 200 mM HEPES and 50 mM NaOH. To each well of a 24-well cell culture plate, 500 µL of collagen-cell solution was added and allowed to polymerize at 37 °C for 1 h. The gels were carefully detached from the well walls before resuspension of gels in 1% FBS-DMEM and maintained at 37 °C, in a 5% CO_2_ atmosphere for 24 h. The DMEM medium was removed and replaced with serum-free DMEM medium with or without 10 ng/mL TGF-β1 and/or 10 µg/mL AF38Pep. Gels were photographed and measured at days 0, 1, 2, 4, and 6 after treatment. The average contraction values were obtained using ImageJ v1.37c (NIH, Bethesda, MD, USA) and are expressed as a percentage of contraction versus the initial gel diameter at 0 days.

### 4.15. Statistical Analysis

For all data with 2 independent variable groupings, two-way analysis of variance (2-way ANOVA) was used, followed by Bonferroni’s multiple comparisons and posthoc Tukey’s test. Graphical data are expressed as means ± S.E. of three independent experiments. All data were analyzed using GraphPad Prism v7 (GraphPad Software, San Diego, CA, USA). Where considered statistically significant, analysis was displayed on figures as: *, *p* ≤ 0.05; **, *p* ≤ 0.01; and ***, *p* ≤ 0.001, or ns if no significance was found.

## Figures and Tables

**Table 1:** Predicted integrin α_4_β_1_/β_7_ amino acids with contact with the EDA-FN peptide.

Mode	Score	Subunit	Integrin Amino Acid in Contact (≤3 Å)
1	−5.0	α_4_	E^121^, D^123^, *N^124^, *K^149^, I^154^, K^155^, *N^156^, E^157^, *N^158^, *K^159^, F^191^, G^192^
		β_1_	V^192^, M^193^, I^196^, S^197^, T^198^, T^199^, P^200^, *A^201^, *K^202^, L^203^, P^206^, C^207^, T^208^, S^209^, N^244^, L^245^, *G^291^, H^312^
		β_7_	V^201^, P^203^, V^205^, S^206^, T^207^, V^208^, P^209^, S^210^, *K^211^, L^212^, P^215^, C^216^, P^217^, T^218^, *N^254^, L^255^, *G^301^
2	−4.4	α_4_	E^121^, D^123^, *N^124^, R^147^, *K^149^, I^154^, K^155^, *N^156^, *K^159^, Y^220^
		β_1_	Y^153^, V^192^, M^193^, T^199^, P^200^, A^201^, *K^202^, L^203^, P^206^, C^207^, T^208^, G^243^, *N^244^
		β_7_	V^201^, P^203^, P^209^, S^210^, *K^211^, L^212^, P^215^, C^216^, P^217^, T^218^, *N^254^, L^255^
3	−3.3	α_4_	I^154^, K^155^, *N^156^, E^157^, N^158^, K^159^, K^190^, F^191^, G^192^
		β_1_	Y^153^, T^199^, P^200^, *A^201^, *K^202^, *R^204^, *N^205^, P^206^, C^207^, T^208^, L^245^
		β_7_	V^201^, P^203^, P^209^, S^210^, *K^211^, L^212^, *R^213^, P^215^, C^216^, T^218^, L^255^
4	−3.1	α_4_	I^154^, N^156^, E^157^, K^159^, K^190^, F^191^, Y^220^, W^221^
		β_1_	S^197^, T^198^, P^200^, A^201^, *K^202^, *R^204^, P^206^, C^207^, T^208^, S^209^, *N^244^, L^245^
		β_7_	V^201^, P^203^, P^209^, S^210^, *K^211^, L^212^, *R^213^, P^215^, C^216^, P^217^, T^218^, G^253^, *N^254^, L^255^
5	−2.6	α_4_	E^121^, D^123^, N^124^, I^154^, K^155^, *N^156^, E^157^, N^158^, K^159^, K^190^, F^191^, G^192^, Y^220^, W^221^
		β_1_	Y^153^, *S^197^, T^199^, P^200^, *A^201^, *K^202^, R^204^, N^205^, P^206^, C^207^, T^208^, G^243^, N^244^
		β_7_	P^209^, S^210^, *K^211^, L^212^, *R^213^, P^215^, C^216^, T^218^, G^253^, N^254^
6	−2.2	α_4_	E^121^, *N^124^, R^147^, I^154^, K^155^, *N^156^, *E^157^, *N^158^, *K^159^
		β_1_	T^191^, V^192^, T^198^, *T^199^, P^200^, A^201^, K^202^, L^203^, T^208^, G^291^, G^292^, H^312^
		β_7_	T^200^, V^201^, L^202^, *T^207^, V^208^, P^209^, S^210^, K^211^, L^212^, T^218^, G^301^
7	+3.3	α_4_	I^154^, K^155^, *N^156^, N^158^, *K^159^, F^191^
		β_1_	*T^191^, V^192^, *S^197^, *T^198^, T^199^, P^200^, *A^201^, K^202^, L^203^
		β_7_	*T^200^, V^201^, *S^206^, *T^207^, V^208^, P^209^, S^210^, *K^211^, L^212^

* indicates predicted H-bond donor.

**Table 2:** Custom primers used for qRT-PCR.

Primer Target	Forward Primer (5′-3′)	Reverse Primer (5′-3′)
hsa_ACTA2	CCGGGACTAAGACGGGAATC	TTGTCACACACCAAGGCAGT
hsa_GAPDH	TCGGAGTCAACGGATTTGGT	TGAAGGGGTCATTGATGGCA
mmu_Acta2	CTGACAGAGGCACCACTGAA	ACAATACCAGTTGTACGTCCAGAG
mmu_Col1a1	CTGGTGAACAGGGTGTTCCT	AAACCTCTCTCGCCTCTTGC
mmu_Col3a1	TGGACCTCCTGGAAAAGATG	GAGCCCTCAGATCCTCTTTCA
mmu_Fn1	ACGGTTTCCCATTACGCCAT	GGCACCATTTAGATGAATCGCA
mmu_Eda-Fn1	GAATCCAGTCCACAGCCATT	TGAACACTGGGTGCTATCCA
mmu_Mmp9	GACTTTTGTGGTCTTCCCCA	AGCGGTACAAGTATGCCTCTG
mmu_Tgfb1	GGCAGCTGTACATTGACTT	CCTTGCTGTACTGTGTGTCC
mmu_Ltbp1	GGGTGTGTGGATGTGAACGA	GCTGACGATCCACACCTGAA
mmu_Hic5	CTATAGCTGGGCAAGTGGTTA	CAAAAGGGAGCCCCATCCTT
mmu_Mrtfa	GCACAGGCATGAGACTGAATTG	AGCTTCAACTGCAGCACTGAC
mmu_Gapdh	AAGAGGGATGCTGCCCTTAC	TACGGCCAAATCCGTTCACA

## Data Availability

The data that support the findings of this study are available from the corresponding author upon reasonable request.

## Supplementary Materials

The following are available online at https://www.mdpi.com/1422-0067/22/4/1575/s1, Figure S1: IST-9 antibody blockade of ERK1/2 and FAK signaling at 12 h post-TGF-β1 treatment. Figure S2: Modeller scores for ITGB1 mapped to the ITGB7 template. Figure S3: PROCHECK pass results for the α_4_β_1_ 3D receptor model. Figure S4: Verify3D pass results for the α_4_β_1_ 3D receptor model.

## Abbreviations

AF38Pep	Antifibrotic 38-amino-acid polypeptide
α-SMA	α-smooth muscle actin
COL	Collagen
Cy5	Cyanine5
ECM	Extracellular matrix
ERK1/2	Extracellular signal-regulated kinases 1 and 2
EDA	Extra domain A
EDB	Extra domain B
EDA-FN	EDA fibronectin
FAK	Focal adhesion kinase
FN	Fibronectin
MMP	Matrix metalloproteinase
OPN	Osteopontin
RGD	Arg-Gly-Asp
TGF-β1	Transforming growth factor-β1
VCAM1	Vascular cell adhesion protein-1
