# Supplementary material for: Design and Evaluation of a Polypeptide That Mimics the Integrin Binding Site for EDA Fibronectin to Block Profibrotic Cell Activity
Source: Int J Mol Sci. 2021 Feb 4;22(4):1575. doi: 10.3390/ijms22041575 (PMC7913925; doi:10.3390/ijms22041575)
Supplement: Supplementary file 1 [file ijms-22-01575-s001.pdf]

Supplementary Materials for

Design and evaluation of a polypeptide that mimics the integrin binding site for EDA fibronectin to block pro-fibrotic cell activity

Lin Zhang[1], Hongyu Yan[1], Yifan Tai[1], Yueming Xue[1], Yongzhen Wei[1], Kai Wang[1], Qiang Zhao[1], Shufang Wang[1,*], Deling Kong[1,2,*] and Adam C. Midgley[1,2,*]

[1] Key Laboratory of Bioactive Materials, Ministry of Education, College of Life Sciences, Nankai University, Tianjin, 300071, China

[2] Rongxiang Xu Center for Regenerative Life Science, State Key Laboratory of Medicinal Chemical Biology, College of Life Sciences, Nankai University, Tianjin, 300071, China

* Correspondence: wangshufang@nankai.edu.cn (S.W.); kongdeling@nankai.edu.cn (D.K.); midgleyac@nankai.edu.cn, Tel.: +86-1562-004-7851 (A.C.M.)

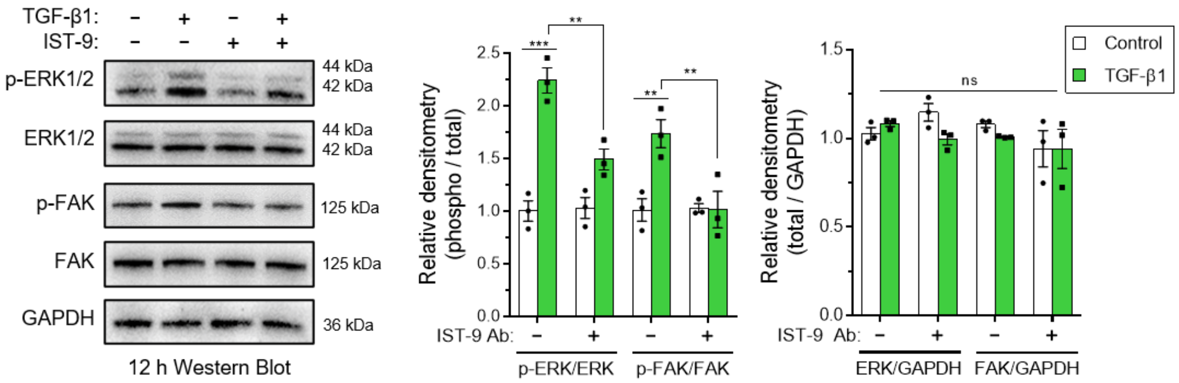

Figure 1. IST-9 antibody blockade of ERK1/2 and FAK signaling at 12 h post-TGF-β1 treatment. NIH/3T3 cells were incubated in the presence or absence of 10 ng/mL TGF-β1 with or without IST-9 antibody for 12 h, before assessment of ERK1/2 and FAK protein expression and phosphorylation. Immunoblots are displayed alongside corresponding densitometry analysis of phospho-protein/total-protein and total-protein/GAPDH loading control. Blots and data are representative of three independent experiments and data is shown as the mean ± S.E. Statistical analysis is shown as $^{***}p \leq 0.001$, $^{**}p \leq 0.01$, ns = no statistical significance ($p > 0.05$).

Model	GA341	zDOPE	Estimated RMSD	Estimated Overlap (3.5Å)
#1.1	1.00	-1.04	1.890	0.937
#1.2	1.00	-1.04	2.211	0.922
#1.3	1.00	-1.05	2.260	0.927
#1.4	1.00	-1.07	1.839	0.932
#1.5	1.00	-1.08	2.158	0.927

Figure S2. Modeller scores for ITGB1 mapped to the ITGB7 template.

```
PROCHECK Summary
Total residues: 966 residues

Ramachandran plot: 81.7% most favoured region
                   17.0% additional allowed region
                   0.7% generously allowed region
                   0.6% disallowed region

All Ramachandrans:     42 labelled residues (out of 955)
Chi1-chi2 plots:       8 labelled residues (out of 583)
Side-chain parameters: 5 better; 0 inside; 0 worse

Residue properties: Max.deviation:  16.2; Bad contacts: 22
                    Bond len/angle: 9.0; Morris et al class: 1 1 2
2 cis-peptides
G-factors Dihedrals: -0.22; Covalent: 0.31; Overall: -0.01

Planar groups: 100.0% within limits; 0.0% highlighted
```

Figure S3. PROCHECK pass results for the $\alpha 4\beta 1$ 3D receptor model.

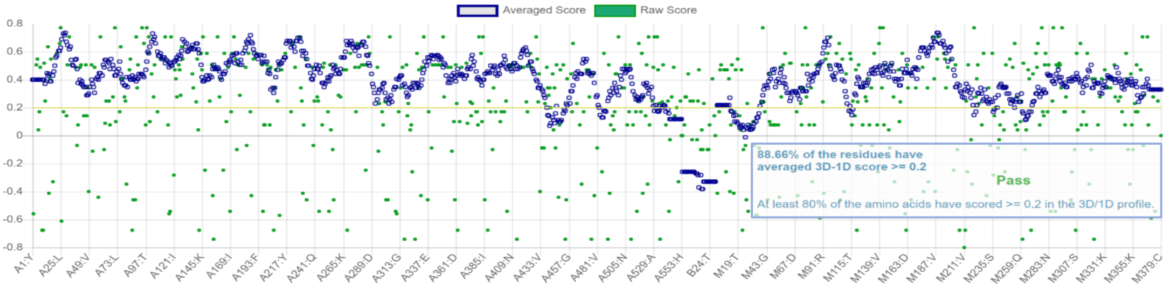

Figure S4. Verify3D pass results for the $\alpha 4\beta 1$ 3D receptor model.
